# Association Between Antibiotic Withdrawal and End-of-Life Comfort in Patients with Advanced Cancer and Suspected Infections: A Multicenter Retrospective Cohort Study

**DOI:** 10.1177/26892820251376993

**Published:** 2025-09-15

**Authors:** Marcela Erazo-Munoz, Diana Catalina Borda Restrepo, Johana Benavides-Cruz, Paloma Beltrán Pachón, Leiner Jesús Colpas Gutiérrez, Jorge Alberto Cortés Luna

**Affiliations:** ^1^ Center for Palliative and Supportive Care, Clinica Reina Sofía, Bogotá, Colombia.; ^2^ Center for Palliative and Supportive Care, Clinica Universitaria Colombia, Bogotá, Colombia.; ^3^ Research Unit, Fundación Universitaria Sanitas, Bogotá, Colombia.; ^4^ Fundación Universitaria Sanitas, Bogotá, Colombia.; ^5^ Clinica Reina Sofía, Bogotá, Colombia.

**Keywords:** antibacterial agents, palliative care, terminal care, patient comfort, terminally ill

## Abstract

**Purpose:**

The role of palliative care in addressing end-of-life needs and enhancing the comfort of patients with life-threatening illnesses is crucial. This study aimed to evaluate the relationship between antibiotic withdrawal and end-of-life discomfort in patients with terminal cancer with suspected infections.

**Materials and Methods:**

A multicenter retrospective cohort study was conducted on patients aged ≥18 years with advanced cancer and life expectancy under six weeks, as determined by a Palliative Prognostic Index >4. Patients were admitted between January 2018 and December 2021, received antibiotics for suspected infections, and died during hospitalization. Patients were categorized into two cohorts based on whether antibiotic therapy was withdrawn or continued within 72–120 hours prior to death. Data were collected through medical chart review. End-of-life comfort was assessed using the validated Edmonton Comfort Assessment Form scale, with scores ≥4 indicating discomfort. Multivariate logistic regression was used to evaluate the association between antibiotic withdrawal and end-of-life discomfort.

**Results:**

A total of 187 patients were included, with a median age of 71 years; 81.8% had solid tumors, predominantly of gastrointestinal origin. Respiratory tract infections were the most common, and sepsis was present in 13% of cases. Symptoms remained mild in both groups, though pain was higher in the antibiotic withdrawal group (median score: 4 vs. 3). Multivariate analysis revealed no significant association between antibiotic withdrawal and end-of-life discomfort (odds ratio = 0.98; 95% confidence interval = 0.52–1.84).

**Conclusions:**

Symptom management for pain, nausea, and dyspnea was generally effective, though moderate pain persisted in some patients after antibiotic withdrawal. Antibiotic withdrawal was not associated with increased discomfort at the end of life. These findings support aligning antibiotic decisions with comfort-focused goals in palliative care.

## Introduction

Palliative care is a cornerstone of the holistic management of patients, particularly for those navigating the challenging end-of-life stage.^1^ Physicians often face ethical dilemmas when deciding whether to withhold or withdraw antibiotic therapy, particularly in patients who lack a treatment escalation limitation plan, that is a strategy to minimize the risk of harm by setting individualized boundaries for treatment in the event of deterioration in the patient’s clinical status.^1–4^ The absence of specific guidance on antibiotic treatment within the existing plan places the onus on the physician to balance the potential benefits and harms associated with antibiotic therapy.^1^ Likewise, achieving a reasonable and comprehensive approach to patients with oncologic pathologies at the end of life requires meticulous consideration of several factors, such as cost–benefit analysis, antibiotic resistance, and adverse effects.^5,6^ In this context, it is important to prioritize symptom relief and comfort, with the crucial goal of reducing infection-related morbidity.^7,8^

In palliative care, infections are often treated empirically since microbiological isolation of pathogens is frequently not performed. Notably, some palliative care patients, especially those with advanced disease or immunocompromised conditions, may experience recurrent infections. In such cases, a key decision in palliative management is assessing the real benefit of initiating antibiotic treatment. This assessment should consider factors such as the patient’s functional status, care goals, and the impact of the treatment on quality of life. Several studies have evaluated the benefits of antibiotics for symptom relief in patients with terminal cancer, finding that antibiotics did not significantly improve symptoms in terminally ill patients.^9–11^ Another study suggests that antibiotics at the end of life may enhance both palliative care and infection management.^12^ In certain scenarios, withholding or withdrawing antibiotics may be an ethically and clinically justified decision. This approach aligns with the principles of proportionality and comfort prioritization, fundamental to palliative care.

In end-of-life care, the Edmonton Comfort Assessment Form (ECAF) is an effective tool for evaluating a broader range of comfort-related issues compared with the Edmonton Symptom Assessment System (ESAS). Designed to provide a more holistic view of patient comfort, the ECAF encompasses physical, emotional, and psychological aspects. It enables health care providers to systematically assess and manage common palliative care symptoms, such as pain, nausea, anxiety, and overall well-being.^13,14^ Additionally, its ease of use and ability to track changes in symptom severity over time make it an invaluable tool for continuous and effective symptom management. This holistic and dynamic approach ultimately enhances patients’ quality of life in their final stages, ensuring they receive the most compassionate and appropriate care possible.^15^

As patients approach the end of life, the benefits of antibiotic use become uncertain, and their administration may lead to adverse effects and contribute to antimicrobial resistance. Because patient priorities often change throughout the course of the terminal illness, it is critical to maintain an ongoing dialogue with patients and their relatives about the benefits and limitations of treatment. When a comfort-focused approach is chosen, it is imperative to prioritize interventions that alleviate suffering and discontinue those that do not directly contribute to the patient’s well-being.^16^ Therefore, the role of antibiotics in symptom relief at the end of life remains unclear. This study aims to evaluate the relationship between antibiotic withdrawal and end-of-life discomfort in terminal cancer patients with suspected infections.

## Materials and Methods

### Study design

This retrospective multicenter cohort study was conducted from January 2018 to December 2021 in three high-complexity hospitals in Bogotá, Distrito Capital, Colombia. In these institutions, patients received inpatient care from their primary medical team in conjunction with the institutional palliative care program. Palliative care in Colombia is still developing, with disparities in access across different regions. The hospitals included in this study are located in major cities and offer outpatient, home care, and hospital services in various specialties. Each hospital has an integrated palliative care program composed of interdisciplinary teams that specialize in caring for people with advanced, chronic, and terminal illnesses. These teams are part of a network whose focus is to provide palliative care services through a unifying, person-centered care model, with the aim of alleviating pain and suffering and improving quality of life. Since 2018, this program has maintained the highest quality and safety standards, as certified by the New Health Foundation.

### Study participants

This study consecutively included patients aged 18 or older with an oncologic diagnosis who were admitted to general hospitalization wards for antibiotic treatment due to a suspected infection. To meet the criteria for end-of-life care, patients were required to have a life expectancy of less than six weeks, as determined by the Palliative Prognostic Index (PPI) score of >4. As demonstrated by Morita et al.,^17^ a PPI score >4 predicts a survival duration of less than six weeks, with a sensitivity of 70% and a specificity of 85%. Exclusion criteria included patients actively undergoing chemotherapy. PPI scoring was performed by palliative care team members as part of the routine clinical evaluation of patients referred to the palliative care program. Patients were categorized into two cohorts based on whether antibiotic therapy was withdrawn or continued within 72–120 hours prior to death.

### Variables

Collected clinical data included age, hospital admission and death dates, oncological diagnosis, presence of delirium (assessed using the Confusion Assessment Method scale), Karnofsky Performance Status Index, and quick SOFA scores at admission. Additionally, ESAS scores for pain, nausea, dyspnea, and well-being, as well as ECAF scores at various time points (upon hospital admission, 72 hours post-admission, and 72 hours prior to death), were recorded. ECAF is an observational scale that is employed in patients who are not able to communicate to evaluate their comfort. It evaluates six situations using a Likert-type scale, ranging between 0 and 4 where 0 means that the condition is not present and 4 means the worst presentation of the condition. The summation of the six items gives a range between 0 and 24 points, 24 representing maximum discomfort and zero representing the maximum possible comfort. Prior research suggests that ESAS and ECAF scores ≥4 indicate inadequate symptom control and comfort, respectively.^15,18^ Information on antibiotic therapy, including the prescription details, duration, number of antibiotics prescribed, and any withdrawal within five days before death. Data on infection characteristics—including the infection site, presence of systemic inflammatory response syndrome (defined as the presence of two or more of the following: (1) fever >38°C or hypothermia <36°C, (2) tachycardia >90 beat/minute; (3) tachypnea >24 breaths/minute; (4) leukocytosis >12,000 cells/mm^3^)—as well as culture results, isolated pathogens, and resistance profiles, were extracted. Data were collected from electronic medical and laboratory records.

### Statistical analysis

Measures of central tendency and dispersion were used to analyze quantitative variables, while qualitative variables were reported as absolute and relative frequencies. The normality of quantitative variables was assessed using the Shapiro-Wilk test. To identify the best model for the association between antibiotic withdrawal and discomfort (measured by the ECAF scale within 72 hours prior to death, with a score of ≥4 indicating discomfort), a backward stepwise multivariate logistic regression was performed, including the following covariates age, sex, pain score, well-being score, nausea score and dyspnea score, infectious focus, tumor type (solid/hematologic), and symptomatic management, which had a *p* < 0.25 in univariate analysis, resulting in a parsimonious model. Model fit was evaluated using the Pearson chi-square goodness-of-fit test. A nonsignificant *p* value was interpreted as an indication that the observed values did not significantly differ from those expected under the model, suggesting an adequate fit. A *p* value <0.05 was considered statistically significant. All analyses were performed using Stata® version 18.

### Ethical considerations

In accordance with resolution 8430 of 1993 issued by the Colombian Ministry of Health and Social Protection, this study was classified as “research without risk.” This study was conducted in line with the principles of the Declaration of Helsinki. Approval was granted by the Ethics Committee of Fundación Universitaria Sanitas under Minute No. 041-21.

## Results

**FIG. 1.**
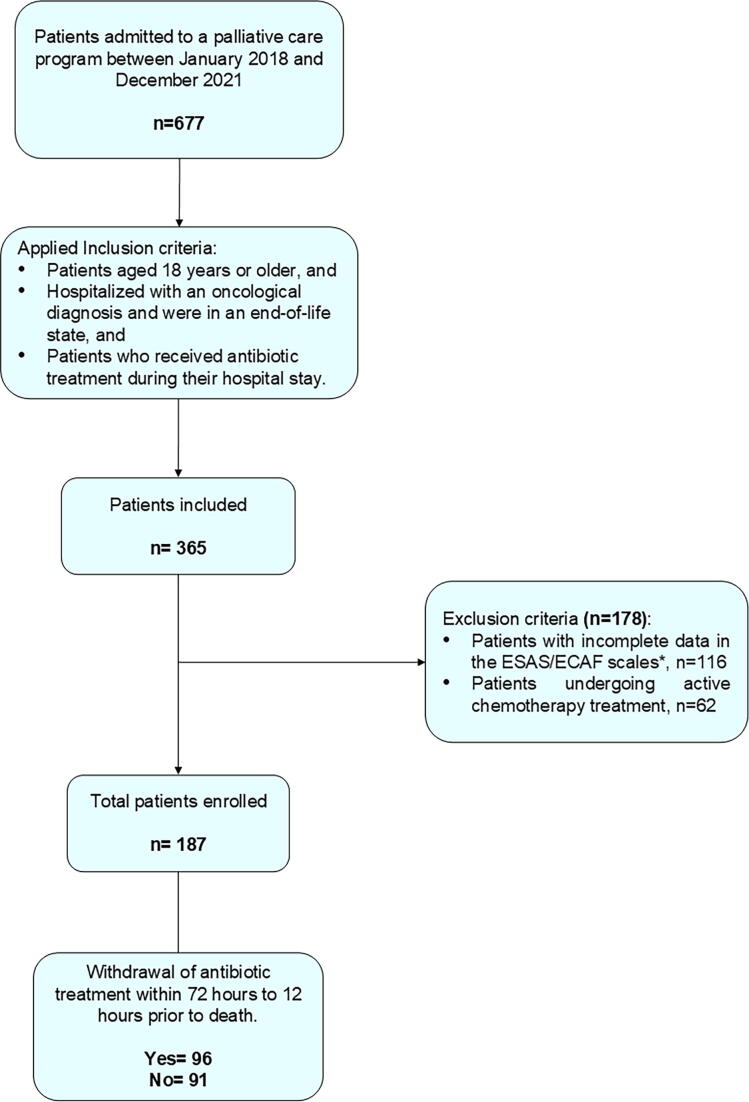
Participant flowchart. ECAF, Edmonton Comfort Assessment Form; ESAS, Edmonton Symptom Assessment System.

A total of 187 patients met the eligibility criteria. Figure 1 illustrates the participant flowchart for the study. The median age of the cohort was 71 years (interquartile range [IQR] = 55–82), with 54.0% of participants being female. Solid tumors were present in 81.8% of the cases, with gastrointestinal and hepatobiliary tumors being the most common (Table 1). Upon admission, respiratory tract infections were identified in 41.2% of patients, followed by urinary tract infections in 28.2%, while sepsis was observed in 13.0% of cases (Table 2). Approximately 27.3% of patients exhibited signs of systemic inflammatory response, with leukocytosis (66.7%) and fever (58.8%) being the most frequently reported indicators. Regarding the number of infectious foci, most patients (92.0%) had a single focus of infection, with a similar distribution observed among those with and without antibiotic withdrawal (Table 2).

**Table 1.** Clinical Characteristics of End-of-Life Patients in the Study

Variable	All (*n* = 187)	No antibiotic withdrawal (*n* = 91)	Antibiotic withdrawal (*n* = 96)
Age (years), median (Q1–Q3)	71 (55–82)	71 (59–84)	70.5 (52–81)
Admission until death (days), median (Q1–Q3)	9 (4–16)	6 (4–11)	12 (7–17)
Karnofsky Index, median (Q1–Q3)	30 (20–50)	40 (30–50)	30 (20–50)
Duration of antibiotic treatment (hours), median (Q1–Q3)	168 (72–288)	120 (72–264)	192 (120–312)
qSOFA score at admission, median (Q1–Q3)	3 (2–3)	3 (2–3)	3 (2–3)
Sex, *n* (%)			
Male	86 (46)	42 (46.2)	44 (45.8)
Female	101 (54)	49 (53.8)	52 (54.2)
Delirium, *n* (%)			
Yes	72 (38.5)	35 (38.5)	37 (38.5)
Oncological diagnosis, *n* (%)			
Hematological tumors	34 (18.2)	16 (17.4)	18 (18.7)
Gastrointestinal tumors	31 (16.6)	18 (19.6)	13 (13.5)
Hepatic, biliary tract, and pancreatic tumors	28 (15.0)	10 (10.9)	18 (18.7)
Gynecological tumors	16 (8.5)	9 (9.8)	7 (7.3)
Breast tumor	14 (7.5)	9 (9.8)	5 (5.2)
Lung tumor	13 (7.0)	6 (6.5)	7 (7.3)
Prostate tumor	11 (5.9)	5 (5.4)	6 (6.3)
Renal and urinary tract tumors	9 (4.8)	5 (5.4)	4 (4.2)
Central nervous system tumors	7 (3.7)	2 (2.2)	5 (5.2)
Head and neck tumors	6 (3.2)	3 (3.3)	3 (3.1)
Skin and soft tissue tumors	8 (4.3)	4 (4.4)	4 (4.2)
Other tumors^a^	10 (5.3)	4 (4.4)	6 (6.3)

^a^ Secondary malignant tumors, thyroid gland tumors, testicular tumors, mediastinal tumors, and bone tumors.

qSOFA, quick SOFA.

**Table 2.** Infection Characteristics and Treatments According to Antibiotic Withdrawal

Variable	All (*n* = 187)	No antibiotic withdrawal (*n* = 91)	Antibiotic withdrawal (*n* = 96)
Infectious focus,^a^ *n* (%)			
Respiratory	77 (41.2)	46 (50.5)	31 (32.3)
Urinary	53 (28.3)	23 (25.3)	30 (31.3)
Gastrointestinal	22 (11.8)	8 (8.8)	14 (14.6)
Intra-abdominal	19 (10.2)	6 (6.6)	13 (13.5)
Soft tissue	17 (9.1)	7 (7.7)	8 (8.3)
Febrile neutropenia	10 (5.3)	7 (7.7)	3 (3.1)
Other infections^b^	6 (3.2)	2 (2.2)	4 (4.0)
Sepsis, *n* (%)			
Yes	24 (13.0)	10 (11.0)	14 (14.6)
Number of infectious foci, *n* (%)			
Single focus	172 (92.0)	83 (91.2)	89 (92.7)
Two foci	15 (8.0)	8 (8.8)	7 (7.3)
Culture taken before the start of antibiotics, *n* (%)			
Yes	92 (49.2)	45 (49.5)	47 (48.7)
Type of culture, *n* (%)			
Blood culture	47 (25.1)	23 (25.3)	24 (25.0)
Urinary culture	65 (34.8)	32 (35.2)	33 (34.4)
Stool culture	4 (2.1)	4 (4.4)	0 (0.0)
Pleural fluid culture	2 (1.1)	2 (2.2)	0 (0.0)
Peritoneal fluid culture	3 (1.6)	1 (1.1)	2 (2.1)
Others^c^	4 (2.1)	2 (2.2)	2 (2.1)
No culture	62 (33.1)	27 (29.7)	35 (36.4)
Antibiotic therapy, *n* (%)			
Monotherapy	90 (48.1)	50 (54.9)	40 (41.6)
Two antibiotics	50 (26.7)	22 (24.2)	28 (29.2)
Three or more antibiotics	47 (25.1)	19 (20.9)	28 (29.2)
Other treatments, *n* (%)			
Non-NSAID analgesics	182 (96.8)	90 (97.8)	92 (100.0)
NSAIDs	77 (41.0)	33 (35.8)	44 (45.8)
Corticosteroids	54 (28.7)	24 (26.1)	30 (31.3)
Antiemetics	104 (55.3)	45 (48.9)	62 (64.6)
Benzodiazepines	119 (63.3)	50 (54.3)	69 (71.9)
Antipsychotics	104 (55.3)	43 (46.7)	61 (63.5)

^a^ A patient may have more than one infectious focus.

^b^ Biliary tract, bacteremia, and/or device-associated infection.

^c^ Sputum cultures, abdominal wall fluid, and neck secretions.

NSAID, nonsteroidal anti-inflammatory.

Upon admission, 17.1% of patients were already receiving antibiotic therapy, with piperacillin–tazobactam being the most administered (32.3%) (Table 3). Cultures were obtained from 49.2% (*n* = 92) of patients before initiating antibiotic therapy; of these, 18.5% (*n* = 17) had two or more cultures taken, and 45.6% (*n* = 42) of the patients had passed away before the results of the cultures were available. The most frequently collected samples were urine cultures (52.0%) and blood cultures (37.0%). The most isolated pathogens included *Escherichia coli* (17.0%), *Klebsiella pneumoniae* (3.0%), and *Staphylococcus aureus* (3.0%), with 30.0% of the isolated pathogens exhibiting some form of resistance (Table 2). In terms of antibiotic therapy, monotherapy was the most prevalent approach, used in 48.1% of patients, followed by the combination of two antibiotics in 26.7% of cases (Table 4).

**Table 3.** Prescription Patterns of Antibiotic Therapy

Variable	*n*	%
Specialty initiating antibiotic therapy (*n* = 187)		
General medicine	65	34.76
Internal medicine	46	24.60
Palliative care	34	18.18
General surgery	9	4.81
Infectious diseases	6	3.21
Hematology	5	2.67
Intensive care	5	2.67
Emergency medicine	5	2.67
Others^a^	12	6.41
Specialty withdrawing antibiotic (*n* = 96)		
Palliative care	62	64.58
Internal medicine	10	10.42
General medicine	9	9.38
Infectious diseases	6	6.25
Intensive care	3	3.13
Others^b^	6	6.25
Antibiotic therapy^c^		
Piperacillin–tazobactam	95	26.1
Ampicillin–sulbactam	38	10.44
Meropenem	30	8.24
Metronidazole	29	7.97
Vancomycin	28	7.69
Cephazolin	23	6.32
Cefepime	20	5.49
Cefuroxime	17	4.67
Ertapenem	13	3.57
Clarithromycin	10	2.75
Linezolid	9	2.47
Fluconazole	9	2.47
Trimethoprim sulfamethoxazole	8	2.20
Clindamycin	6	1.65
Ceftriaxone	6	1.65
Ciprofloxacin	6	1.65
Oxacillin	5	1.37
Amoxicillin	4	1.10
Others^d^	8	2.20

^a^ Urology, oncology, pulmonology, neurology.

^b^ Hematology, neurosurgery, general surgery.

^c^ A patient may have more than one prescribed antibiotic.

^d^ Amikacin, cephalexin, amoxicillin clavulanate, doripenem, avibactam + ceftazidime, albendazole, cephalothin, ampicillin.

**Table 4.** Microorganisms Isolated in Urine and Blood Cultures and the Frequency of Antibiotic Resistance

Variable	*n*	%
Urine culture (*n* = 65)		
Positive	46	70.77
Negative	18	27.69
No report	1	1.54
Isolated pathogens		
*Escherichia coli*	16	44
*Klebsiella pneumoniae*	4	11
*Enterococcus faecalis*	4	11
*Pseudomonas aeruginosa*	3	8
Suggestive of contamination	2	6
Others^a^	2	6
Not reported	1	3
Antibiotic resistance		
Yes	15	42
Blood culture (*n* = 46)		
Positive	20	42
Negative	25	56
No report	1	2
Isolated pathogens		
*E. coli*	5	25
*K. pneumoniae*	3	15
*Staphylococcus aureus*	3	15
*Staphylococcus hominis*	2	10
*P. aeruginosa*	2	10
Others^b^	5	25
Antibiotic resistance		
Yes	5	25

^a^ *Citrobacter youngae*, *Klebsiella oxytoca*.

^b^ *E. faecalis*, *Listeria monocytogenes*, *Pantoea dispersa*, *Rhodotorula glutinis/mucilaginosa*, *Serratia marcescens*.

Regarding the specialty responsible for initiating antibiotic therapy, general medicine accounted for 34.8% of prescriptions, followed by internal medicine (24.6%) and palliative care (18.2%). The most used antibiotics were piperacillin–tazobactam (26.1%), ampicillin–sulbactam (10.4%), and meropenem (8.2%) (Table 3), with a median duration of antibiotic therapy of 168 hours (7 days). Antibiotic withdrawal was carried out in 51.3% of patients, predominantly on the recommendation of palliative care (65.3%), followed by internal medicine (10.5%) and general medicine (9.5%) (Table 3). Following the withdrawal of antibiotic therapy, 7.3% (*n* = 7) of patients showed signs of a systemic inflammatory response. The median number of days from hospital admission to death for the entire cohort was 9 days (IQR = 4–16).

During hospitalization, nausea and dyspnea were observed to be mild and adequately controlled in both groups upon admission. However, pain levels differed between the groups, with mild pain reported in patients who did not undergo antibiotic withdrawal, and moderate pain in those who did. At 72 hours after admission, pain, nausea, and dyspnea remained mild in both groups. Similarly, at 72 hours prior to death, nausea and dyspnea continued to be mild, while pain remained mild in the nonwithdrawal group and moderate in the withdrawal group. Throughout the study period, well-being from admission to 72 hours prior to death was not adequately controlled in either group. Comfort was eventually achieved in the entire cohort before death, although it was not well-controlled at admission or 72 hours afterward (Table 5). Adjuvant medications used for symptom control included analgesics (98.0%) and antiemetics (56.1%). The most used analgesics were hydromorphone (25.0%), acetaminophen (22.0%), and hyoscine (18.0%), while the predominant antiemetics were ondansetron (58.0%) and metoclopramide (24.0%) (Table 2).

**Table 5.** Symptom and Comfort Scores of End-of-Life Patients on Admission, 72 Hours Post-Admission, and 72 Hours Predeath

	Upon hospital admission			72 hours post-admission			72 hours predeath		
	All (*n* = 187)	No antibiotic withdrawal (*n* = 91)	Antibiotic withdrawal (*n* = 96)	All (*n* = 187)	No antibiotic withdrawal (*n* = 91)	Antibiotic withdrawal (*n* = 96)	All (*n* = 187)	No antibiotic withdrawal (*n* = 91)	Antibiotic withdrawal (*n* = 96)
Variable	Scores median (Q1–Q3)			Scores median (Q1–Q3)			Scores median (Q1–Q3)		
Pain	4.0 (1.0–7.0)	3.0 (0.0–6.0)	4.0 (2.0–7.0)	3.0 (0.0–4.0)	0.0 (0.0–6.0)	3.0 (1.0–3.0)	3.0 (2.0–6.0)	3.0 (0.0–4.0)	4.0 (3.0–7.0)
Nausea	0.0 (0.0–2.0)	0.0 (0.0–2.0)	1.0 (0.0–4.0)	0.0 (0.0–2.0)	0.0 (0.0–3.0)	0.5 (0.0–1.0)	0.0 (0.0–2.0)	0.0 (0.0–2.0)	2.0 (0.0–3.0)
Dyspnea	0.0 (0.0–3.0)	0.0 (0.0–5.0)	0.0 (0.0–3.0)	0.0 (0.0–2.0)	0.0 (0.0–4.0)	1.5 (0.0–2.0)	3.0 (0.0–4.0)	3.0 (0.0–5.0)	2.5 (0.0–4.0)
Well-being	5.0 (4.0–6.0)	5.0 (4.0–6.0)	5.0 (4.0–6.0)	4.0 (4.0–5.0)	4.0 (4.0–5.0)	4.0 (4.0–5.0)	4.0 (3.0–6.0)	4.0 (3.0–5.0)	4.5 (4.0–6.0)
Comfort	4.0 (1.0–7.0)	3.0 (2.0–7.0)	4.0 (1.0–7.0)	3.0 (0.0–5.0)	4.0 (1.0–11.0)	2.0 (0.0–4.0)	2.0 (0.0–5.0)	2.0 (0.0–5.0)	2.0 (0.0–5.0)

Multivariate logistic regression analysis did not yield statistically significant results, likelihood ratio χ^2^(3) = 3.2, *p* = 0.357, with a pseudo-*R*^2^ of 0.015, indicating limited explanatory power of the model. Antibiotic withdrawal at the end of life was not significantly associated with discomfort (ECAF score ≥4) (Table 6). Model fit was evaluated using the Pearson’s χ^2^ goodness-of-fit test, which yielded a nonsignificant result (χ^2^ = 129.1, *p* = 0.209), suggesting that the model provides an adequate fit to the data.

**Table 6.** Multivariate Logistic Regression

Variable	OR	Standard error	*p* Value	95% CI
Withdrawal of antibiotics	0.98	0.314	0.954	0.52–1.84
Age	0.99	0.009	0.205	0.97–1.01
Presence of SIRS	1.20	0.441	0.628	0.58–2.46

Likelihood ratio χ^2^(3) = 3.23, *p* = 0.357; pseudo-*R*^2^ = 0.015. CI, confidence interval; OR, odds ratio; SIRS, systemic inflammatory response syndrome.

## Discussion

Currently, there is no clear clinical consensus on the treatment of infections in end-of-life care. However, antimicrobial use remains widespread among patients hospitalized in their final days. This study found that the withdrawal of antibiotic therapy in hospitalized patients with advanced oncologic disease was not significantly associated with discomfort at the end of life, as measured by the ECAF. Although patients in the antibiotic withdrawal group reported slightly higher pain levels, especially near the time of death, these differences did not reach statistical significance. Overall, comfort was ultimately achieved in both groups prior to death, suggesting that the withdrawal of antibiotic therapy was not related to inadequate symptom control.

Bhat et al.^19^ analyzed bacterial infection patterns in patients with cancer undergoing anticancer treatment, identifying respiratory infections as the most prevalent (28.6%). Similarly, other studies have reported a high frequency of respiratory infections among end-of-life patients with cancer, with prevalence rates reaching up to 50.0%.^11,20–27^ The incidence of sepsis in this population also varies, with up to one-third of patients developing the condition.^11,20,22,23,28^ Our findings are partially consistent with these observations: respiratory tract infections were also the most common infection site (41.2%), whereas sepsis was identified in a smaller proportion of patients (13.0%). This lower proportion may be partly explained by the fact that cultures were not obtained in all patients, potentially limiting microbiological identification and the clinical diagnosis of sepsis.

The primary reasons for antibiotic prescription, as noted by several authors, include managing infection-related symptoms, such as fever, leukocytosis, and pain, or responding to sepsis.^22,23,26–30^ In this study, about one-third of patients exhibited signs of systemic inflammatory response, and 7% developed inflammatory symptoms after antibiotic withdrawal. Sesma et al.^21^ reported that approximately 8.0% of patients received antibiotic treatment before hospitalization. In contrast, our study found that nearly double that percentage of patients had received antibiotic treatment within 15 days prior to their current hospital admission. Cultures were obtained from around 50.0% of patients, consistent with findings by Lopez et al.^20^ Notably, nearly half of the patients who underwent culture testing died before the results were available. Most studies indicate that cultures are conducted in nearly all patients with cancer at the end of life,^22–24,28^ with urine and blood cultures being the most common. However, limited information is available regarding the patients’ condition when culture results are received.

In the present study, the most frequently isolated pathogens in urine samples were *E. coli*, *K. pneumoniae*, and *Enterococcus faecalis*, whereas in blood samples, *E. coli*, *K. pneumoniae*, and *S. aureus* were primary pathogens. These findings are consistent with previous studies that have documented a similar pattern.^21,31^ However, other studies have shown variations, such as one identifying *S. aureus* was the most frequent pathogen^28^ and another in which *Klebsiella* spp. was predominant,^19^ suggesting that pathogens’ distribution may differ based on study settings and geographic region. Murray et al.^31^ reported that these pathogens are among the top six pathogens responsible for more than 250,000 deaths associated with antimicrobial resistance. In the present study, 30.3% of the pathogens exhibited some form of antimicrobial resistance, result that is consistent with other studies, where resistance rates ranged from 2.0% to 83.0%, depending on the microorganism and its resistance profile.^19,28^ This great variability may be explained by specific characteristics of the study population, the type of health care institution, local microbiological patterns, and prior exposure to antimicrobials. These findings highlight the importance of considering bacterial resistance when making therapeutic decisions, even in end-of-life care.

The most used antibiotics were piperacillin–tazobactam, ampicillin–sulbactam, and meropenem. This finding is consistent with existing literature, which shows that beta-lactams, carbapenems, and cephalosporins are frequently administered in end-of-life care, with empirical monotherapy being the predominant approach.^20–22,26–28,32^ The median duration of antibiotic therapy in this population was seven days, which is shorter than the 10- to 18-day range reported in previous studies.^11,25^ No study has directly compared patients who continued antibiotic therapy until death with those in whom it was withdrawn. However, one study compared patients who did and did not receive antibiotics at the end of life, finding no statistically significant differences in documented symptoms or hospital length of stay.^20^ Studies focused on antibiotic withdrawal indicate that the time from discontinuation to death is typically less than one week.^21,23,25^

The empirical use of broad-spectrum antimicrobials in end-of-life patients remains a topic of ongoing debate. Despite the advanced disease stage and multiple comorbidities of these patients, antibiotics are frequently administered without clear evidence of infection, raising questions about their necessity and benefit.^22,33,34^ In many cases, the potential risks, such as side effects, prolonging the dying process, and delaying the transition to palliative or hospice care, are not fully considered.^35^

It is not uncommon for physicians to prescribe antibiotics in response to infections without adequately considering the patient’s prognosis. This practice is often driven by a desire to offer hope to the patient and their family or a sense of acting, even when the intervention may not align with the patient’s best interests or therapeutic goals.^33^ The findings of this study indicate that general practitioners and internal medicine specialists were the most frequent prescribers of antibiotics. Conversely, palliative care specialists were more likely to discontinue their use when therapeutic objectives were not met, considering continued use to be a futile intervention.

The lack of a statistically significant association in the multivariate analysis (pseudo-*R*^2^ = 0.015) suggests that factors other than antibiotic use play a more prominent role in determining end-of-life comfort. These findings are consistent with the existing literature^20,23,24,29,36^; however, direct comparisons remain challenging due to variations in clinical assessment tools across studies. While antibiotics are often used for symptom relief or to prolong survival, their benefits are diminished when the patient’s prognosis is limited to just days. In such cases, clinicians face difficult decisions regarding when to withdraw antibiotics. In line with the principle of respecting patient autonomy, withdrawal decisions should prioritize the patient’s wishes when they have the capacity to agree.^2^ When patients lose this capacity, the medical team must make decisions based on factors such as the risk–benefit ratio and the patient’s best interests, with quality of life, comfort, and symptom control becoming the primary goals.

This study has several limitations that should be considered with interpreting the results. First, due to its retrospective design, it cannot infer causality between antibiotic withdrawal and symptoms. Additionally, it may introduce information bias because symptoms and comfort assessments were based solely on medical and nursing records, precluding the possibility of direct observation. Nevertheless, this study used standardized symptoms and comfort assessment scales, which helped to obtain reliable, comparable data. Second, the scales used in this study are specific to palliative care services, and their completion is mandatory for patients evaluated by these services. Consequently, patients not enrolled in the palliative care program were excluded, as well as those receiving palliative care for noncancer diagnoses, which limits the generalizability of the results to other populations. Third, the lack of microbiological confirmation in all patients limits the ability to precisely determine the infectious etiology, potentially leading to misclassification of suspected infections. Fourth, cultural and institutional factors, as well as patient characteristics or physician preferences, may influence decisions regarding antibiotic use and end-of-life care, limiting the generalizability of our findings to other settings. Last, all patients received pharmacologic management for symptom control, making it difficult to determine whether antibiotic withhold or withdrawal directly affected end-of-life comfort.

In conclusion, symptom management for pain, nausea, and dyspnea was generally effective. However, moderate pain persisted in some patients, especially those who had undergone antibiotic withdrawal. The administration of analgesics and antiemetics likely contributed to overall comfort. Conversely, multivariate logistic regression analysis indicated that antibiotic withdrawal was not associated with end-of-life comfort, suggesting that discontinuing antibiotics in this context does not appear to exacerbate symptoms, as these symptoms were adequately managed with symptomatic treatment. The findings support the use of tailored antibiotic strategies and emphasize the role of timely symptom management to optimize patient comfort at the end of life.

As is well known, the goals of palliative care are related to comfort and the improvement of quality of life, as well as involving patients and families in decision making and clearly communicating goals of care. While these principles should already guide end-of-life care, our findings highlight the need to strengthen their consistent application, particularly in the context of antimicrobial use. This study reinforces the importance of establishing regular evaluations—focused on symptoms, comfort, and quality of life—to reassess the appropriateness of continuing antibiotic treatment. These evaluations support more deliberate and individualized decisions, helping to ensure that care remains aligned with patient-centered goals.

## Data Availability

The data that support the findings of this study are not openly available due to reasons of sensitivity and are available from the corresponding author upon reasonable request.

## Abbreviations Used

ECAF: Edmonton comfort assessment form
ESAS: Edmonton symptom assessment system
PPI: Palliative prognostic index
